# Editorial: Ethnic inequalities in diabetes care and outcomes

**DOI:** 10.3389/fcdhc.2025.1595078

**Published:** 2025-05-09

**Authors:** Suma Uday

**Affiliations:** Department of Endocrinology and Diabetes, Birmingham Women’s and Children’s Hospital, Birmingham, United Kingdom

**Keywords:** diabetes, ethnicity, glycated (glycosylated) haemoglobin, socioeconomic, children

Health is defined as a state of complete physical, mental and social well-being and not merely the absence of disease or infirmity. Inequalities in health resulting in differences in health outcomes arise from social, economic, environmental, and structural disparities. Health inequalities are universally seen in all societies from low to middle to high income countries. The societal health inequalities were more evident and exacerbated by the COVID-19 pandemic with higher numbers of COVID-19 related cases and deaths seen in areas of higher socioeconomic disadvantage and among minority ethnic groups in the Western world. Health inequalities in diabetes care are widespread and impact on all aspects from prevention to access to technology/treatment to morbidity and mortality. The intention of this Research Topic was to throw light on the existing inequalities and to move forward with solutions for tackling some of these inequalities.

The varied respected contributors to this topic bring to light several key aspects of health inequalities in diabetes care. With diabetes being one of the most rampant non-communicable disease, prevention must be a key aspect of management. Frigerio et al. elegantly summarise in a mini-review the role of neighbourhood inequalities on diabetes prevention in high income countries. The review highlights that diabetes prevention and care is affected at a multidimensional level in the presence of disadvantaged neighbourhood factors such as socioeconomic status, food environment, walkability and neighbourhood aesthetics. For instance, walkability, greenspace presence and air quality in neighbourhoods were correlated with reduced diabetes incidence and prevalence. The role of neighbourhood deprivation on access to basic and novel anti-diabetic medications along with access to healthcare services related to T2DM is noteworthy. The authors rightly conclude that addressing individual factors alone is not sufficient to tackle the problem, especially in the most deprived cohorts. A call for policymakers to develop evidence-based policies at national and regional levels to implement change at the population level is justified.

It is well recognised that the ethnic minority groups in developed countries are disproportionality affected by health inequalities particularly by higher diabetes risk and poorer outcomes. Supported self-management programmes have been effective in positively influencing glycaemic control and lifestyle modifications. The effectiveness of such programmes in ethnic minority groups in developed counties is less clear, therefore, Grant and Litchfield explore the role of Community Health Worker and Peer supporter or educator (CHWP) led interventions designed to improve self-management of type 2 diabetes (T2D) within ethnic minority groups in a systematic review. The authors provide clarity by summarising the findings under a modified framework encompassing five domains of Affective attitude, Burden and Opportunity Costs, Cultural Sensitivity, Intervention Coherence and Effectiveness and Self-efficacy. The authors found that the building of a trusting relationship by the CHWPs with the patients through a culturally sensitive approach encouraged personalised care and improved overall patient experience although some concerns were raised about the lack of clinical knowledge in CHWPs. The universally known barriers such as lack of attendance or engagement were also noted. A range of factors relating to personal circumstances (poor health, work, logistical barriers to travel) and the cost of fresh food impacted engagement with the intervention. The authors concede that addressing these concerns requires close working with the local government or healthcare services which in turn warrants broader consideration at a health economics and policy level. One of the implications on future practice suggested by the authors was the need for CHWPs to ideally speak the same native language as participants to combat barriers of language and (health) literacy which is a key concept addressed by Idkowiak et al. in their single centre retrospective review. Idkowiak et al. explore glycaemic control at 18 months following diagnosis in a multi-ethnic cohort of children and young people with type 1 diabetes (T1D), comparing outcomes in children and families who require an interpreter (INT, n=41) vs those who don’t (CTR, n=100). Despite the CTR group having a higher HbA1c at baseline the INT group had a poorer HbA1c at 18 months. The INT group were also noted to be predominantly from a more deprived background which adds to the burden. The authors highlight that diabetes specific training of interpreters may help improve outcomes alongside language concordant care. Improving care in the deprived cohort of children requires a multi-dimensional approach including improved access to healthcare, a theme that resonates across all the articles in the Research Topic.

Mondkar et al. take a slightly more clinical approach to the topic and report on the inequalities seen with regards to insulin resistance in adolescents with T1D in the Indian sub-continent. In an attempt to improve glycaemic outcomes in adolescents with T1D and suspected metabolic syndrome adjunctive therapy is tried. The authors report the effect of metformin at 9 months on glycaemic control, insulin sensitivity (IS), cardiometabolic parameters and body composition in 89 Indian adolescents with T1D in a randomised, double-blind, placebo-controlled trial. Metformin adjunct therapy in Asian Indian adolescents with T1D demonstrated a favourable effect on glycaemic control, glycaemic variability, insulin sensitivity, lipid profile, vascular function, body mass index and body fat composition with a good safety profile. Previous studies have demonstrated similar effect. The optimum duration of therapy however remains to be determined.

Finally, in the Research Topic, Dickinson et al. report on technology usage and glycaemic outcomes in 222 children with T1D from a single tertiary centre in the UK. The UK and several other developed countries demonstrate a significant difference in HbA1c among the native and immigrant populations in favour of the natives. The study centre differs from the rest of the UK population in terms of demographics and covers a predominantly ethnic minority (60%) and socioeconomically deprived (60%) cohort. Uptake of technology in the deprived cohort reassuringly improved glycaemic control with the best outcome seen in those using hybrid closed loop systems. Nonetheless, the use of these technologies was higher in the most affluent groups and the authors push for use of advanced technologies in the disadvantaged groups who will benefit most. Interestingly, whilst equalising technology access reduced socioeconomic disparities in HbA1c, ethnic disparities persisted. The authors note that individuals of Black ethnicity continued to have a higher HbA1c. The authors speculate a residual glucose-independent effect which has previously been recorded in other reports but not explored in detail and therefore warrants further investigation.

Collectively, these articles throw light on the broad range of health inequalities and how these impact on diabetes prevention, care and outcomes. It is very evident that the ethnic minority and deprived cohorts suffer the most and a positive change requires multi-dimensional approach from addressing individual factors to neighbourhood to institutional elements and broader policy matters.

